# Das Tübinger Modell der „Ethikbeauftragten der Station“: Ein Pilotprojekt zum Aufbau dezentraler Strukturen der Ethikberatung an einem Universitätsklinikum

**DOI:** 10.1007/s00481-021-00635-0

**Published:** 2021-06-17

**Authors:** Robert Ranisch, Annette Riedel, Friedemann Bresch, Hiltrud Mayer, Klaus-Dieter Pape, Gerda Weise, Petra Renz

**Affiliations:** 1 Klinisches Ethik-Komitee, Universitätsklinikum Tübingen, Gartenstraße 47, 72074 Tübingen, Deutschland; 2 Professur für Medizinische Ethik mit Schwerpunkt auf Digitalisierung, Fakultät für Gesundheitswissenschaften Brandenburg, Universität Potsdam, Potsdam, Deutschland; 3 Institut für Ethik und Geschichte der Medizin, Eberhard Karls Universität Tübingen, Tübingen, Deutschland; 4 Fakultät Soziale Arbeit, Bildung und Pflege, Hochschule Esslingen, Esslingen, Deutschland; 5 Klinik für Kinder- und Jugendmedizin, Universitätsklinikum Tübingen, Tübingen, Deutschland; 6 Katholische Klinikseelsorge in der Psychiatrie und Neurologie, Universitätsklinik Tübingen, Tübingen, Deutschland; 7 Stroke Unit und neurologische Intensivüberwachung, Neurologische Klinik, Universitätsklinikum Tübingen, Tübingen, Deutschland; 8 Stabsstelle Vorstand KV 4 – Pflegeorganisation Qualitätsentwicklung und Pflegeberatung, Universitätsklinikum Tübingen, Tübingen, Deutschland

**Keywords:** Klinisches Ethik-Komitee, Ethikberatung, Klinische Ethik, Pflegeethik, Ethische Fallbesprechung, Organisationsethik, METAP, Clinical ethics committees, Clinical ethics consultation, Clinical ethics, Nursing ethics, Moral case deliberation, Organizational ethics, METAP

## Abstract

Ethik-Komitees gehören zum festen Bestandteil des Ethikmanagements und der Organisationsethik in klinischen Einrichtungen des Gesundheitswesens. Entsprechende Ethikstrukturen und die damit verbundenen Angebote stoßen hinsichtlich ihrer Wirksamkeit allerdings an ihre Grenzen. Ihre Arbeitsweisen sind häufig reaktiv und eine Verankerung in den entsprechenden Organisationsebenen fehlt. Ausgehend von diesen Limitationen der klinischen Ethikberatung hat sich die multiprofessionelle „Arbeitsgruppe Ethik“ am Universitätsklinikum Tübingen (UKT) um die Konzeption und Implementierung eines neuen Ansatzes zur nachhaltigen Integration von ethischen Reflexions- und Entscheidungsprozessen auf den Stationen des UKT bemüht. Mit dem Tübinger Modell der Ethikbeauftragten der Station verfolgt sie ein Pilotprojekt, das speziell geschulte Pflegekräfte aus allen Stationen des UKT als AnsprechpartnerInnen für ethische Fragen einsetzt. Damit stellen die Ethikbeauftragten eine Erweiterung zu etablierten Strukturen der Ethikberatung dar und ergänzen vorhandene Top-Down-Strategien. Der vorliegende Beitrag stellt die Zielsetzungen des Tübinger Modells dar und schildert erste Erfahrungen in der Umsetzung. Neben der Einbettung in organisationale Strukturen der Ethikberatung werden die stationsinternen und stationsübergreifenden Aufgaben der Ethikbeauftragten dargestellt. Zudem wird das Qualifikationsprogramm für Ethikbeauftragte (Basis- und Aufbauschulung) sowie ein Train-the-Trainer-Konzept vorgestellt, welche eine vertiefende Entwicklung von pflege- und medizinethischer Kompetenzen unterstützen und Sicherheit in den stationsbezogenen Reflexions- und Entscheidungsprozessen vermitteln.

## Abstract (en)

**Definition of the problem:**

Clinical ethics committees form an integral part of ethics management and organizational ethics in clinical healthcare facilities. However, working mostly reactive and not being anchored in the corresponding organizational levels, such instruments are limited in terms of effectiveness.

**Arguments:**

Based on these shortcomings of clinical ethics consultation the multiprofessional working group “Ethics” at the University Hospital Tübingen, Germany has endeavored to design and implement new structures for sustainable ethical decision-making processes on its hospital wards. The Tübingen Model “Ethics Consultants on the Ward” is a pilot project intended to appoint specifically trained nursing staff on all wards as contact partners for ethical issues. Thus, the Tübingen Model represents an extension to established structures of clinical ethics consultation and complements existing top-down strategies.

**Conclusions:**

This article presents the objectives of the Tübingen Model and describes initial experiences in its implementation. After explaining their role within the existing structures of ethics consultation at the hospital, the tasks of the ethics consultants within and across the hospital’s wards are presented. Furthermore, the qualification program for ethics consultants (basic and advanced training) as well as a train-the-trainer concept are presented, which support an in-depth development of competencies in nursing as well as medical ethics and provide confidence in the reflection and decision-making processes on the ward.

## Einleitung

Strukturen der Ethikberatung haben sich an Deutschlands Kliniken in den letzten drei Jahrzehnten zunehmend etabliert (Dörries und Hespe-Jungesblut 2007; Schochow et al. 2019). Diese Entwicklung folgte einem internationalen Trend, der in den 1980er-Jahren in den USA mit der Einrichtung von klinischen Ethik-Komitees begann (Frewer 2008) und zuweilen sogar als „Ethikboom“ beschrieben wird. Angesichts neuer Behandlungsmöglichkeiten, Entwicklungen in der Intensiv- oder Transplantationsmedizin, wachsender Sensibilisierung gegenüber ethischen Fragen und nicht zuletzt auch aufgrund von Zertifizierungsmöglichkeiten stieg der Bedarf an Ethikmanagement in Einrichtungen des Gesundheitswesens zunehmend (zum Überblick: Dörries et al. 2010; Frewer et al. 2008, 2012; Schildmann et al. 2010; Simon 2020).

Mittlerweile liegen eine Reihe von Empfehlungen für die Gestaltung von entsprechenden Strukturen vor. Die Zentrale Ethikkommission (ZEKO) bei der Bundesärztekammer veröffentlichte bereits 2006 eine Stellungnahme zur Ethikberatung in der klinischen Medizin (ZEKO 2006). Seitens der Akademie für Ethik in der Medizin (AEM) wurden 2010 Standards für die Ethikberatung in Einrichtungen des Gesundheitswesens vorgeschlagen (AEM 2010), die als maßgebend in Deutschland gelten können. Hinzu kommen eine ganze Reihe von Modellen und Methoden zur ethischen Fallbesprechung bzw. Fallberatung, die nunmehr in Einrichtungen des Gesundheitswesens Anwendung finden (exemplarisch: Albisser Schleger et al. 2019; Marckmann 2015; Reiter-Theil 2005; Steinkamp und Gordijn 2010; Riedel und Lehmeyer 2016).

Das Thema der Ethikberatung wird auch mit Blick auf Aspekte der Organisationsethik erforscht (Heinemann und Maio 2010; Krobath und Heller 2010). Entsprechende Fragestellungen begegnen dem Thema hier aus unterschiedlichen Perspektiven: So kann nach ethischen Konfliktfeldern gefragt werden, die sich auf Ebene der Organisation stellen. Seitens der ZEKO wurden etwa zur Klärung von sensiblen Finanzierungs- oder Allokationsentscheidungen Organisationsethik-Komitees vorgeschlagen – ein Impuls, der bislang allerdings nicht aufgegriffen wurde. Organisationsethische Fragen können sich zudem hinsichtlich der geeigneten Strukturen einer erfolgreichen Ethikberatung stellen, also hinsichtlich der Organisation von Ethik. Hierzu gehören Aspekte eines verantwortlichen Ethikmanagements in Einrichtungen des Gesundheitswesens sowie Fragen der Implementierung von entsprechenden Strukturen, Maßnahmen und handlungsleitenden Instrumenten.

Für die erfolgreiche Umsetzung von Ethikberatung wird gemeinhin eine Verzahnung von „oberer“ und „unterer“ Organisationsebene vorgeschlagen (Vollmann 2010). Die Standards der AEM formulieren hierzu: „Voraussetzungen für eine erfolgreiche Ethikberatung sind die Verankerung in der Mitarbeiterschaft und die Unterstützung durch die Leitungsebene“ (AEM 2010, S. 150). Eine solche Kombination von Bottom-up- und Top-down-Elementen bildet sich auch in Ansätzen zum Ethikmanagement in Kliniken ab, etwa im klinisch-ethischen Interaktionsmodell von Norbert Steinkamp und Bert Gordijn (2010, S. 139–140) sowie anderen Vorschlägen (Albisser Schleger et al. 2019).

Während der „Ethikboom“ in der Klinik als grundsätzlich begrüßenswert eingeschätzt wird, fehlt es allerdings nicht an Kritik an den bislang institutionalisierten Maßnahmen. So stellen Meinolfus W. M. Strätling und Beate Sedemund-Adib zumindest den hierzulande dominierenden Werkzeugen der Ethikberatung, den klinischen Ethik-Komitees sowie der ethischen Fallberatung, ein denkbar schlechtes Zeugnis aus:

So wohlmeinend und langfristig potenziell interessant das Gesamtprojekt „klinische Ethikberatung“ auch erscheint, muss davon ausgegangen werden, dass zumindest bisher entsprechende „Serviceeinrichtungen“ in der Regel nicht in der Lage sind, einen robusten und glaubwürdigen Anspruch zu erheben, ausgerechnet bei besonders schwierigen Zweifels- und Konfliktfällen kompetent beratend, geschweige denn vermittelnd oder gar entscheidend tätig zu werden. (Strätling und Sedemund-Adib 2013, S. A828)

Die Ethik in der Klinik würde demnach weit hinter einstigen Erwartungen zurückstehen, keine nötige Akzeptanz haben und folglich auch selten in Anspruch genommen werden. Der „Ethik-Community“ sei es bislang nicht gelungen, durch „Leistung, Qualität und Kompetenz“ zu überzeugen. Einen Grund hierfür sehen die AutorInnen auch in verfehlten Organisationsstrukturen: Anstelle der Förderung von Ethikkompetenz *in den* Heilberufen, würden ethische Herausforderungen an „Experten“ delegiert, die häufig „vollkommen fachfremd sind oder sich der klinischen Tätigkeit und Verantwortung weitgehend entfremdet haben“ (Strätling und Sedemund-Adib 2013, S. A826). Selbst Autoren, die der benannten Ethik-Community zugerechnet werden können, sehen zumindest in der bisherigen Umsetzung von Ethik in der Klinik einige Defizite. So gesteht Ralf Jox in einer Replik auf Strätling und Sedemund-Adib (2013) ein, dass es der Ethikberatung häufig an Ressourcen und Vernetzung fehle. Sie müsse zudem ihre Sichtbarkeit erhöhen, sich niederschwelliger und bedarfsorientierter anbieten und Instrumente der Qualitätsmessung etablieren (Jox 2014).

Es kann lohnen, am Projekt der klinischen Ethikberatung festzuhalten und neben Ethik-Komitee und ethischen Fallbesprechungen neue Wege zu bemühen, Ethik in der Klinik zu organisieren. Dieses Ziel verfolgt das *Tübinger Modell der Ethikbeauftragten der Station*, welches seit 2020 am Universitätsklinikum Tübingen erprobt wird. Damit wird die Absicht verfolgt, mittelfristig auf allen Stationen des Klinikums eine/n ausgebildete/n MitarbeiterIn zu stellen, der/die als AnsprechpartnerIn für ethische Belange fungiert und zugleich eine Schnittstelle zu zentralen Organisationseinheiten der Ethikberatung (insb. dem Klinischen Ethik-Komitee) bildet. Der vorliegende Artikel stellt das Tübinger Modell dar und schildert die Konzeptualisierung, das Qualifizierungsprogramm und erste Erfahrungen der Implementierung.

## Ethikberatung am Universitätsklinikum Tübingen (UKT)

Das Universitätsklinikum Tübingen (UKT) gehört mit seinen 1585 Betten zu den größten Einrichtungen des Gesundheitswesens in Baden-Württemberg und beschäftigt ca. 10.000 MitarbeiterInnen in 17 Kliniken, 17 Zentren und 15 Instituten. Jährlich versorgt es mehr als 74.000 stationäre sowie 380.000 ambulante PatientenInnen.

Das UKT hat sich in Deutschland früh mit der Etablierung von Strukturen der klinischen Ethikberatung beschäftigt (zur Geschichte: Marckmann und Wiesing 2008). Erste Impulse reichen auf eine klinisch-ethische Gesprächsrunde zurück, die in den 1980er-Jahren initiiert wurde. Im Jahr 1998 wurde an der Universität Tübingen der deutschlandweit erste Lehrstuhl für Ethik in der Medizin eingerichtet. Auf Initiative von MitarbeiterInnen wurde 2004 durch den Vorstand des Klinikums ein multiprofessionell besetztes Klinisches Ethik-Komitee (KEK) eingerichtet. Das noch heute bestehende KEK besteht aus 18 Mitgliedern und tagt turnusmäßig viermal pro Jahr sowie bei Bedarf. Gegenwärtig zählen zu den Mitgliedern VertreterInnen unterschiedlicher Bereiche der Medizin, Pflege und Physiotherapie, der Seelsorge, Medizinethik, Medizinrecht sowie der Pflegedirektor und die kaufmännische Direktorin des Klinikums.^1^ Die Leitung des Gremiums wird durch zwei Vorsitzende sowie eine Geschäftsführung wahrgenommen. Vorrangiges Ziel des KEK ist die Bereitstellung einer Infrastruktur für die Ethikberatung. Hierzu gehören ethische Fallbesprechungen, die Entwicklung von Leitlinien für wiederkehrende ethische Herausforderungen sowie die Organisation und Durchführung von Fort- und Weiterbildungen (AEM 2010).

Überdies haben sich verschiedene Arbeitsgruppen des KEK formiert, die aus (ehemaligen) Mitgliedern, assoziierten oder themenrelevanten VertreterInnen der Klinikabteilungen bestehen. Hierzu zählt etwa die Arbeitsgruppe „Ethik in der Pflege“ (kurz: AG Ethik), die auf Impulse von MitarbeiterInnen der Pflege und Seelsorge zurückgeht. Seit 2007 plant und organisiert die AG Ethik den „Ethiktreff“ – ein vier- bis sechsmal jährlich stattfindendes Forum mit Weiterbildungscharakter. Die AG Ethik berichtet dem KEK über ihre laufenden Aktivitäten sowie den Austausch mit Pflegekräften.

Zu den Kernaufgaben des KEK gehört die ethische Fallbesprechung im Einzelfall. Auf Anfrage werden kurzfristig Teambesprechungen in den jeweiligen Stationen durchgeführt. Entsprechende Anfragen werden durch die Geschäftsführung des KEK koordiniert und von ausgebildeten Mitgliedern der AG „Ethikberatung“ moderiert, dokumentiert und im KEK berichtet bzw. nachbesprochen. Alle MitarbeiterInnen des Klinikums sowie PatientInnen und Angehörige können entsprechende Ethikkonsile anfragen. Ziel der Fallbesprechungen ist eine möglichst multiperspektivische Betrachtung der ethischen Konfliktsituation, die in einem strukturierten Gesprächsprozess idealiter konsensual aufgelöst werden kann. Aus diesem Grund sind Fallbesprechungen am UKT überaus inklusiv und umfassen nicht selten mehr als 10 Parteien, Gesprächsgruppen aus klinischem Personal sowie Angehörige und PatientInnen. Die Zahl an Anfragen liegt gegenwärtig bei ca. 30 pro Jahr.

## Limitationen der Ethikberatung und Hinderungsgründe der Inanspruchnahme

Obgleich die Akzeptanz und Qualität der klinischen Ethikberatung am UKT als gut einzuschätzen ist, wurde im KEK sowie der AG Ethik kontinuierlich über mögliche Hindernisse bei der Wahrnehmung des Beratungsangebots diskutiert. Dabei konnte eine Reihe von Faktoren identifiziert werden, die auf persönlichen Gesprächen mit MitarbeiterInnen, dem Austausch im „Ethiktreff“ sowie den Erfahrungen aus einer fünfzehnjährigen Praxis der Ethikberatung beruhen:

- *Zielgruppe*: Per Satzung ist das KEK Ansprechpartner für alle MitarbeiterInnen des Klinikums, für PatientInnen ebenso wie für Angehörige. Die Praxis der ethischen Fallbesprechung zeigt allerdings, dass der weit überwiegende Teil von Anfragen auf das ärztliche Personal zurückgeht. Der langjährige Austausch mit Pflegekräften im „Ethiktreff“ gibt anekdotische Evidenz über bestehende Hemmnisse seitens des nichtärztlichen Personals, insbesondere der Pflegekräfte, das ethische Beratungsangebot anzufragen.
- *Reichweite*: Durch Broschüren, Informations- und Weiterbildungsveranstaltungen sowie Auftritte im Inter- und Intranet wird am Klinikum über Maßnahmen der Ethikberatung informiert. Erfahrungen zeigen allerdings eine mitunter geringe Bekanntheit der Angebote des Ethik-Komitees sowie von Funktion und Ablauf der Ethikkonsile. Hierzu passend zeigt sich ein Muster an wiederkehrenden Anfragen von Stationen, die bereits in der Vergangenheit das Beratungsangebot nutzten.
- *Einstiegshürde*: Anfragen nach Fallbesprechungen stehen in aller Regel mit akuten und drängenden Konfliktsituationen in Verbindung. Dies erschwert präventive Maßnahmen, um entsprechende Konstellationen vorzubeugen. Zudem beschränken sich Anfragen häufig auf den akutmedizinischen Bereich. Vermeintlich weniger drängende ethische Fragestellungen in anderen Bereichen der Patientenversorgung werden anekdotisch berichtet, führen allerdings kaum zur Inanspruchnahme der unterstützenden Angebote im Rahmen der Ethikberatung.
- *Zeit- und Ressourcenintensität*: Da ethische Fallbesprechungen häufig als *ultima ratio* in Akutsituationen in Anspruch genommen werden, sind entsprechende Konfliktsituationen häufig komplex. In der Folge gestalten sich die Ethikberatungen mitunter als ein zeit- und ressourcenintensives Unterfangen für die jeweiligen Stationen. Bei vermeintlich weniger komplexen, dennoch konfliktträchtigen ethischen Fragestellungen besteht vielfach eine Hemmung der Inanspruchnahme der Unterstützung.
- *Verankerung*: Die ethische Fallbesprechung erfolgt als fallbezogene Teambesprechung auf der jeweiligen Station, die durch qualifizierte Mitglieder der AG Ethikberatung moderiert werden. Während dies die Neutralität und Qualität der Fallbesprechung unterstützt, erschwert der „externe“ Zugang den kontinuierlichen Austausch mit den Stationen. Über die jeweilige Fallbesprechung hinaus kann das Fallgeschehen selten begleitet werden. Follow-ups oder Rückmeldungen über Ausgang und Qualität der Ethikkonsile erfolgen häufig nur informell.

Die Erfahrungen am UKT decken sich zum Teil mit Untersuchungen zu möglichen Hinderungsgründen, Maßnahmen der Ethikberatung in Einrichtungen des Gesundheitswesens in Anspruch zu nehmen (Tab. 1) bzw. erfolgreich zu implementieren (Ranisch und Brand 2016). Mögliche Faktoren können sowohl auf Ebene der Organisationsformen (z. B. Ressourcenmangel), der beteiligten Personen (z. B. allgemeine Skepsis gegenüber externer Beratung) oder der Ethikberatungsleistung selbst (z. B. mangelnde Qualität) verortet werden. In der Fachliteratur werden zudem berufsgruppenspezifische Hemmnisse unterschieden: So äußerten ÄrztInnen etwa Bedenken gegenüber möglichen Einmischungen in die PatientInnen-Beziehung, der fachlichen Kompetenz und Qualifikation von EthikberaterInnen sowie dem Nutzen entsprechender Angebote (Dörries 2003; Isaacs 2018; Pedersen et al. 2009; DuVal et al. 2004; Gaudine et al. 2011; Orlowski et al. 2006). Seitens der Pflegekräfte wird dagegen von Sorgen berichtet, mit Inanspruchnahme von Ethikberatung bestehende Hierarchien zu umgehen (Gaudine et al. 2011). Eine gesteigerte Bereitschaft gegenüber dem Angebot scheint zudem in Verbindung zu stehen mit bestehenden Bekanntschaften zu Personen aus Ethik-Komitees (Gaudine et al. 2011).

**Table 1:** 

Fehlende Kenntnis über Angebot, Aufgabe, Erreichbarkeit, Ablauf oder Funktion von Ethikberatung bzw. fehlende Sichtbarkeit derselben
Fehlende Erfahrung oder Sensibilität im Umgang mit ethischen Konfliktsituationen
Sorge um negative Resonanz des Kollegiums oder um mögliche Missachtung von Hierarchien
Zweifel am Nutzen, der Qualität, Validität oder Transparenz der Ethikberatung
Zweifel an der Qualifikation und Kompetenz der EthikberaterInnen sowie Sorge vor „Praxisferne“
Fehlende Fehlerkultur, mangelnde Unterstützung bei der Wahrnehmung von Ethikberatung
Überschätzung der eigenen Fähigkeiten bzw. professionellen Rolle
Ressourcenintensität bzw. mangelnde personelle oder zeitliche Mittel
Skepsis gegenüber einer Öffnung „nach außen“ bzw. gegenüber externen Beratern
Wahrnehmung als Feigenblatt, PR- oder bloße Zertifizierungs-Maßnahme
Bürokratische oder administrative Hürden
Sorge vor Einmischung bei Behandlungsentscheidungen oder Kompetenzverlusten
Sorge vor moralischer Anklage
Fehlendes Zuständigkeitsgefühl bzw. Verantwortungsdiffusion bzgl. eines potenziellen Falls
Sorge über Moralisierung oder religiöse Einflussnahme

## Das Tübinger Modell der „Ethikbeauftragten der Station“

Die Erfahrungen am UKT sowie die berichteten Hürden bei der Wahrnehmung von klinischer Ethikberatung wurden zum Anlass genommen, weitergehende Möglichkeiten der Implementierung von Strukturen der Ethikberatung zu bedenken. Diese sollten sowohl die Qualität und Unabhängigkeit der bestehenden Angebote wahren sowie durch neue Maßnahmen Hemmnisse der Nutzung von Ethikberatung reduzieren. Im Ergebnis wurde in einem Pilotmodell am Universitätsklinikum Tübingen ein neuer Ansatz des Ethikmanagements konzipiert, implementiert und (zukünftig) evaluiert: das *Tübinger Modell der Ethikbeauftragten der Station*.

Der Impuls für die Entwicklung des Tübinger Modells ging von der AG Ethik aus, die in einer Unterarbeitsgruppe Möglichkeiten diskutierte, die Ethikkompetenz insbesondere der Pflegekräfte zu fördern und den Austausch über ethische Belange in den jeweiligen Stationen des Klinikums zu stärken.^2^ Nach einer Phase der Ideenfindung wurde die Arbeitsgruppe 2016 durch die Pflegedirektion des Klinikums mit der Entwicklung eines Konzeptpapiers betraut. Zwischen 2016 und 2019 wurden in regelmäßigen Planungstreffen durch Mitglieder der AG Ethik, der Geschäftsführung des Ethik-Komitees sowie der Stabsstelle Pflegeorganisation des Universitätsklinikums Zielsetzungen und Maßnahmen zum Ausbau des Ethikmanagements formuliert und mit relevanten KlinikvertreterInnen diskutiert.

Die Konzeptentwicklung erfolgte im Austausch mit den TeilnehmerInnen des „Ethiktreff“, die Impulse für mögliche Verbesserungen der Organisationsstruktur des Ethikangebots am UKT gaben. Im Planungsteam wurden zudem bestehende Ansätze und Modelle eines umfassenden Ethikmanagements recherchiert und erfasst, die in deutschsprachigen Einrichtungen der Patientenversorgung eingesetzt werden. Dabei hatte insbesondere METAP (Albisser Schleger et al. 2019) eine Vorbildwirkung für das Tübinger Modell. Dieses knüpft damit zugleich an verschiedene Bemühungen in ausländischen Gesundheitseinrichtungen an, ethisches Denken und Handeln als Teil der gesamten Organisationskultur zu fördern, Strukturen zur Verknüpfung zwischen den etablierten Formaten der Ethikberatung und den jeweiligen Organisationseinheiten aufzubauen, und damit ein Wertebewusstsein in der alltäglichen Praxis der Gesundheitsberufe zu stärken (exemplarisch: Bates et al. 2017; MacRae et al. 2005; Fox et al. 2010).

### Zielsetzungen des Tübinger Modells

Primäre Zielsetzung des Tübinger Modells stellt die verbesserte Organisation von Ethik am Universitätsklinikum dar. Für diesen Zweck werden dauerhafte, dezentrale Strukturen geschaffen, die das bestehende Angebot einer zentralen Ethikberatung durch das Ethik-Komitee bzw. die AG Ethikberatung ergänzen sollen. Zu den Organisationseinheiten des Tübinger Modells gehören i. eine verstetigte Projektstelle für die Ethikbeauftragten, ii. ein Pool an EthiktrainerInnen sowie iii. die Ethikbeauftragten auf den jeweiligen Stationen. Als initiale Zielgruppe stehen die professionell Pflegenden des Klinikums im Mittelpunkt des Vorhabens. Dies einerseits als Ethikbeauftragte, die in ihrer Rolle ethische Fragestellungen identifizieren und die Angebote der Ethikberatung aufzeigen sowie koordinieren. Andererseits geht es auch darum, die Hemmschwelle innerhalb der Berufsgruppe der Pflegenden zu reduzieren, professionsbezogene Konflikte zu benennen und belastende ethische Konfliktsituationen zur Sprache zu bringen.

Durch ein Qualifizierungsprogramm für Pflegekräfte soll deren ethische Sensibilität unterstützt und diese bekräftigt werden; ihre ethischen Wahrnehmungs- und Entscheidungskompetenzen sollen gefördert werden. Auf den jeweiligen Stationen des Klinikums sollen zudem Routinen institutionalisiert werden, die den Austausch über ethische Fragen nicht auf Akutsituationen begrenzen und – in Form des zentralen Angebots einer klinischen Ethikberatung – an Dritte übertragen, sondern regelhaft in den Arbeitsalltag integrieren. Das Tübinger Modell möchte somit zugleich Hinderungsgründe umgehen, die der Ethikberatung in Einrichtungen des Gesundheitswesens im Wege stehen.

Die vorläufige Fokussierung auf die Berufsgruppe der Pflegekräfte war durch verschiedene Faktoren bedingt: Aus Perspektive der Organisationsentwicklung sind sie als größte Berufsgruppe am Klinikum naheliegender Adressat für ein integratives Ethikangebot. Da das ärztliche Personal häufig im Rotationsprinzip Stationen des Klinikums wechselt, die Ethikbeauftragten allerdings als kontinuierliche Ansprechpartner fungieren sollen, hatte die Qualifizierung von Pflegekräften während der Pilotphase des Tübinger Modells zudem Vorrang und wurde auch im Sinne einer Professionalisierung der Pflege aktiv durch die Pflegedirektion in Abstimmung mit dem Klinikumsvorstand unterstützt. Aufgrund der häufig gegebenen Nähe in der Patientenversorgung sind Pflegekräfte überdies eng in ethisch sensible Situationen eingebunden. Damit in Verbindung stehen zugleich Belastungserlebnisse, die nicht nur auf Personal- oder Ressourcenmangel, sondern auch auf anhaltenden moralischen Stress („*moral distress*“) zurückgeführt werden können und nicht selten zum Berufsausstieg führen – eine langanhaltende Entwicklung, die sich im Zusammenhang mit der COVID-19-Pandemie noch einmal verschärft.

Als sekundäre Zielsetzung des Tübinger Modells gilt daher die Reduzierung von moralischem Stress („*moral distress*“) des Pflegepersonals und anderer MitarbeiterInnen. Es wird hypothetisiert, dass die routinemäßige Verhandlung von ethisch aufgeladenen Situationen im Behandlungsteam die Risikofaktoren für moralischen Stress (Corley et al. 2001; Epstein und Hamric 2009) verringert und schließlich auch zur Arbeitszufriedenheit beiträgt. Begleitend zur Implementierung wird daher eine Prä-Post-Erhebung zur Wahrnehmung und Relevanz ethischer Konflikte im Stationsalltag sowie zum Erleben von moralischem Stress durchgeführt (siehe Abschnitt „Evaluation“). Dies soll zugleich eine Evaluation dieses erweiterten Angebots der Ethikberatung erlauben. Durch die Stärkung von Ethikkompetenz auf den Stationen sowie durch die Gewährleistung eines regelhaften Austauschs über entsprechende Themen im Behandlungsteam wird zudem ein positiver Effekt auf die Zufriedenheit von PatientInnen sowie Angehörigen wie auch in Bezug auf den Erhalt der Versorgungsqualität erhofft.

### Aufgaben der Ethikbeauftragten und Vernetzung zum KEK

Ethikbeauftragte der Station (EBS) sind MitarbeiterInnen, die nach einer mehrtägigen Schulung durch sogenannte EthiktrainerInnen auf ihrer Station als AnsprechpartnerInnen und Bezugspersonen für ethische Fragen fungieren. Die EBS erfüllen dabei sowohl *stationsinterne* als auch *stationsübergreifende *Aufgaben. Im Rahmen ihrer Tätigkeit auf Station sollen sie ethische Handlungsbereiche identifizieren, das Stationsteam für entsprechende Fragestellungen sensibilisieren und dabei unterstützen, dass diese regelhaft zur Sprache kommen. Die EBS moderieren hierzu auch „kleine“ Fallbesprechungen im Behandlungsteam, die sogenannten FiBs. Diese stellen eine niederschwellige Ergänzung zu den etablierten ethischen Fallbesprechungen durch die AG Ethikberatung des KEK dar. Aus ihrer Stationserfahrung sollen EBS zudem einen möglichen Weiterbildungsbedarf im Kollegium hinsichtlich Themen der klinischen Ethik bzw. der Pflegeethik identifizieren und an die Projektstelle für die Ethikbeauftragten bzw. an die EthiktrainerInnen kommunizieren. Entsprechende Impulse werden in fortlaufenden (Aufbau‑)Schulungen oder im Weiterbildungsangebot für die EBS aufgegriffen.

Die EBS sind während der Arbeitstätigkeit die ausgewiesenen Ansprechpersonen für ethisch sensible Entscheidungssituationen auf ihren jeweiligen Stationen. Um ethische Konfliktsituationen frühzeitig zu identifizieren, soll eine regelhafte Kommunikation bspw. einmal wöchentlich in der Übergabe von Früh- auf die Spätschicht oder in jeder Teamsitzung erfolgen. Dabei liegt der thematische Fokus auf der stationären Versorgung der PatientInnen, insbesondere hinsichtlich ethischer Fragen einer möglichen Über‑, Unter- oder Fehlversorgung, Entscheidungskonflikten mit Angehörigen oder bspw. wenn die Pflegekraft die Patientenautonomie als gefährdet einstuft. Strukturell zu klärende Fragen (z. B. Unterbesetzung des Pflegeteams und daraus resultierende Überlastanzeigen) sollen nicht in diesem Rahmen besprochen werden.

Die EBS sollen so (potenzielle) ethisch sensible Vorkommnisse wahrnehmen, ansprechen und gemäß definierter Entscheidungsroutinen entsprechende Schritte in die Wege leiten. Die Bearbeitung von ethischen Konfliktsituationen erfolgt dabei in Anlehnung an das vierstufige Eskalationsmodell von METAP (Albisser Schleger et al. 2019, S. 229–238) und sieht folgende Routine vor: 1. Individuelle Entscheidungsfindung der Betroffenen mit dem/der EBS, 2. Beratung mit den EthiktrainerInnen, 3. Fallbesprechung im Behandlungsteam (FiBs) sowie 4. Inanspruchnahme einer ethischen Fallbesprechung durch das Ethik-Komitee. Bevor ein Ethikkonsil ausgelöst wird, werden Konfliktsituationen also zunächst auf den Stationen adressiert: durch Austausch mit den EBS, den EthiktrainerInnen bzw. durch eine Besprechung der MitarbeiterInnen auf den Stationen.

Ähnlich wie METAP verfolgt das Tübinger Modell damit das Anliegen, ein mehrstufiges Beratungsangebot zu initiieren, welches auf ein Zusammenspiel von unterschiedlichen Organisationsebenen im Klinikum setzt. Das Tübinger Modell weicht dabei nicht nur hinsichtlich der Zielgruppe der Pflegekräfte und dem für sie entwickelten Ausbildungsprogramm von METAP ab, sondern hat den Algorithmus des Eskalationsmodells (Albisser Schleger et al. 2019, S. 234) an gegebene Organisationsstrukturen und -erfordernisse angepasst. Die EBS sowie die MitarbeiterInnen der zentralen Projektstelle erfüllen dabei im Wesentlichen die Aufgaben der in METAP vorgesehenen Steuergruppe der jeweiligen Abteilungen. Bereits die erste Stufe der im Tübinger Model vorgesehenen Entscheidungsroutine ist dialogisch ausgerichtet, sodass im Fall einer potenziellen Konfliktsituation ein kontinuierlicher Austausch zwischen den Betroffenen und einer festen Bezugsperson in Form der/des jeweiligen EBS erfolgt. In der zweiten Stufe wird der in METAP vorgesehene Austausch mit einem Mitglied der Steuergruppe durch eine kollegiale Besprechung mit den EthiktrainerInnen der übrigen Stationen ersetzt. Anders als in der dritten Stufe des METAP Eskalationsmodells möchte die Fallbesprechung im Behandlungsteam (FiBs) im Tübinger Modell bewusst kein übliches Ethikkonsil abbilden und somit bekannte Hemmnisse bei der Wahrnehmung einer zeit- und ressourcenintensiven ethischen Fallbesprechung reduzieren. Die FiBs bilden vielmehr eine niederschwellige Routine ab, bei der ethische Aspekte des Arbeitsalltags regelmäßig auf den Stationen zur Sprache kommen dürfen und sollen. Erst in der letzten Stufe wird eine ethische Fallbesprechung initiiert, wofür die bestehenden Angebote der AG Ethikberatung des KEK genutzt werden. Durch den anhaltenden Austausch zwischen EBS, EthiktrainerInnen und Projektstelle sowie zwischen Projektstelle und dem KEK wird das Beratungsangebot dabei zugleich in die Breite der Organisation getragen.

Die EBS erfüllen so auch klinikumsweit eine Scharnierfunktion zwischen den jeweiligen Stationen und dem Klinischen Ethik-Komitee. Die EBS arbeiten hierzu in einem engen Verbund mit der Projektstelle der Ethikbeauftragten sowie den EthiktrainerInnen, die bei Bedarf weitere kollegiale Unterstützung durch EthiktrainerInnen vermittelt. Die EBS dokumentieren zudem aufkommende und sich wiederholende ethische Fragestellungen und berichten diese in den monatlichen Vernetzungstreffen der EBS aller Stationen. Über die Teilnahme der EBS an den regelmäßig stattfindenden Vernetzungstreffen können diese zugleich Informationen über laufende Bemühungen des Ethikmanagements am Klinikum erfahren und an ihr Kollegium weitergeben sowie ihre Praxiskenntnisse und -erfahrungen austauschen und reflektieren. Eine Teilnahme der EBS am „Ethiktreff“ dient unter anderem der Vertiefung in der Wahrnehmung ethischer Frage- und Problemstellungen – oftmals konkret anhand eines Falles – sowie dem berufsgruppenübergreifenden Austausch. Zugleich berichten Mitglieder der „AG Ethik“ bzw. die Mitarbeiterinnen der Projektstelle in jeder Sitzung des Klinischen Ethik-Komitees über den aktuellen Projektstand und Erfahrungen zu den Aktivitäten auf den Stationen der EBS und stehen in regelmäßigem Kontakt mit der Geschäftsführung des Klinischen Ethik-Komitees. Durch diese Verknüpfung von Bottom-up- und Top-down-Elementen soll ein effektives Ethikmanagement am Universitätsklinikum gefördert werden.

### Besetzung der EBS und Kompetenzanforderung

Am UKT verantworten eine Bereichsleitung und deren Stellvertretungen fachlich und disziplinarisch alle nichtärztlichen Versorgungs- und Organisationsprozesse in einer zugewiesenen Pflege- bzw. Versorgungseinheit. Entsprechend der im Klinikum verankerten Tätigkeitsbeschreibungen liegt die Aufgabe des/der EBS im Verantwortungsbereich der stellvertretenden Bereichsleitung II. Er/Sie hat die Möglichkeit, diese Tätigkeit selbst zu übernehmen oder das Zeitkontingent für die Erfüllung der Aufgaben an eine Pflegekraft des Bereichs zu übertragen.

Die Übernahme der Tätigkeit des/der EBS erfolgt dabei stets freiwillig. Ein Interesse an ethischen Fragen sowie die Bereitschaft zur Weiterqualifikation wird vorausgesetzt. Eine mindestens fünfjährige Erfahrung im Fachbereich sollte gegeben sein, ebenso wie ein Maß an Kommunikations‑, Reflexions- und Durchsetzungsfähigkeit sowie eine bestehende Vertrauensbasis im Kollegium der jeweiligen Station.

### Ressourcenausstattung und Implementierung

Im Vorfeld der Projektumsetzung wurde ein Ressourcenplan erstellt, der den Zeitbedarf der Qualifizierungsmaßnahmen (Basis- und Aufbauqualifizierung) beinhaltet sowie die monatlichen Vernetzungstreffen und kollegialen Beratungen, Fortbildungen und die Aufgabenwahrnehmung auf den jeweiligen Stationen. Der Ressourcenbedarf umfasst im ersten Implementierungsjahr 200 h pro EthiktrainerIn sowie 250 h pro Mitglied der AG Ethik. Um eine kontinuierliche Projektumsetzung und -evaluation zu gewährleisten, wurde eine Projektstelle für das Tübinger Modell mit einem Beschäftigungsumfang von 50 % an der Pflegedirektion etabliert, die fachlich von der Gruppenleiterin der Stabsstelle Qualitätsentwicklung und Pflegeberatung der Pflegedirektion begleitet wird. Die hauptamtlichen Mitarbeiterinnen der Stelle sind fachabteilungsübergreifend tätig. Zu den Aufgaben gehören insbesondere die Struktur- und Qualitätsentwicklung bspw. Projektevaluation, Fortbildungen und Öffentlichkeitsarbeit.

Mittelfristig sollen ausgebildete EBS das Ethikmanagement des Klinikums auf allen bettenführenden Stationen unterstützen. Zur Implementierung wurde ein Roll-out-Plan mit 5 Phasen definiert: Phase 1 beginnt an den „Hot-Spots“, den Intensivstationen und eng angrenzenden Stationen. Phase 2 widmet sich peripheren Stationen in räumlicher Nähe der Stationen in Phase 1. Die in Phase 1 qualifizierten MitarbeiterInnen agieren zugleich als Multiplikatoren und entlasten die EthiktrainerInnen während der nachfolgenden Implementierungsphasen. In Phase 3 und Phase 4 werden weitere periphere Stationen qualifiziert. Phase 5 ist als übergreifende Qualifizierung geplant.

Die Implementierungsphase des Tübinger Modells ist für eine Zeitdauer von vier Jahren angelegt (2020–2024). Die Anzahl der EBS pro Station ist abhängig von der Anzahl der Betten auf Station. Die interne Festlegung beinhaltet für Intensivstationen eine/n EBS pro 20 Betten und für (periphere) Stationen eine/n EBS pro 30 Betten. Pro Station wird mindestens ein/e EBS qualifiziert, so dass insgesamt 60 bis 80 EBS qualifiziert werden.

## Qualifizierungsprogramm und Vernetzung

Das Ausbildungsprogramm des Tübinger Modells umfasst zum einen Qualifizierungsmaßnahmen für die EthiktrainerInnen, zum anderen ein Ausbildungsprogramm der EBS durch die qualifizierten EthiktrainerInnen sowie regelmäßige Aufbauschulungen. Hinzu kommen ein kollegiales Begleitprogramm für die EBS und regelmäßige Vernetzungstreffen.

### Qualifizierungsmaßnahme der EthiktrainerInnen

Die Ausbildung der EBS erfolgt durch EthiktrainerInnen. Diese wurden in einem dreitägigen Train-the-Trainer-Workshop durch eine professionelle Trainerin auf die Qualifizierung der EBS vorbereitet. Die Trainingsinhalte dieses Workshops orientierten sich am Curriculum „Ethikberatung im Gesundheitswesen“ der Akademie für Ethik in der Medizin (2019) sowie den Richtlinien der Schweizerischen Akademie der Medizinischen Wissenschaften (2019) „Ethikausbildung für Gesundheitsberufe“.

Diese beiden Rahmenwerke dienten als Bezugspunkte für die inhaltliche Ausgestaltung der dreitägigen Qualifizierung wie auch zur Festlegung der zu erwerbenden Kompetenzen seitens der TeilnehmerInnen. Neben den Ethikkompetenzen der zukünftigen EthiktrainerInnen stand parallel der Erwerb von methodischen und didaktischen Kompetenzen im Fokus der Qualifizierungsmaßnahme. Die Realisierung der geplanten Methoden diente zugleich der Validierung der zukünftigen Einbindung in die eigenen Schulungen.

Folgende Lehr- und Lerninhalte standen bei der Qualifizierung der EthiktrainerInnen im Mittelpunkt:

1. Grundlagen der Ethik
2. Bedeutung ethischer Reflexion als Gegenstand professionellen Handelns
3. Relevanz der systematisierten ethischen Reflexion und Entscheidungsfindung
4. Ethische Fallbesprechung inklusive Übungen
5. Strukturierte Methode der ethischen Entscheidungsfindung
6. Eskalationsstufen in Anlehnung an das Modell METAP
7. Kompetenzen und Aufgaben der TrainerInnen und der EBS

Die zuvor klar definierten Kompetenzen der zukünftigen EBS, die seitens der qualifizierten EthiktrainerInnen ausgebildet werden sollen, rahmten die Ausrichtung der Trainer-Qualifizierung im Sinne dessen, dass sie klarlegen, welche Kompetenzen es zukünftig zu entwickeln und zu vertiefen gilt, welche Kompetenzen zu antizipieren und deren Entwicklung es methodisch zu unterstützen gilt.

Sowohl die Kompetenzen der EBS wie auch die Lehr- und Lerninhalte der zukünftigen Qualifizierung durch die EthiktrainerInnen und die zukünftige Performanz der EBS bildeten in der Schulung der TrainerInnen den konzeptionellen, inhaltlichen und methodisch-didaktischen Rahmen, stets verbunden mit den Zielperspektiven:

- Lehr- und Lerninhalte didaktisch ansprechend und praxisorientiert zu vermitteln
- diese (Ethik‑)Kompetenzen als EthiktrainerIn methodisch zu unterstützen, zu entwickeln und zu verdichten
- den Erwerb der Ethikkompetenzen methodisch unterstützt zu evaluieren (summativ und formativ)

### Qualifizierungsmaßnahme der Ethikbeauftragten der Station

Die EBS durchlaufen eine zweitägige Basisschulung sowie eine zusätzliche Aufbauqualifizierung. Diese werden seitens der qualifizierten EthiktrainerInnen realisiert. Die erste Schulungseinheit von EBS erfolgte in Begleitung der Trainerin der EthiktrainerInnen und diente zugleich einer weiteren Validierung und Evaluation der Lehr- und Lerninhalte, der methodischen Ausgestaltung und der didaktischen Konzeption der beiden Schulungstage für die EBS. Nach der ersten Schulungseinheit wurde das Schulungskonzept konsentiert. Es beschreibt die folgenden zu erwerbenden (Ethik‑)Kompetenzen der zukünftigen EBS:

- Ethische Fragestellungen identifizieren, abgrenzen, analysieren und reflektieren können
- Das Bewusstsein für die existenzielle Dimension ethischer Fragen und Konflikte
- Die Sensibilität für die Vielfalt an situativ wirkenden Werten, Normen und Perspektiven
- Förderliche Rahmenbedingungen für die ethischen Abwägungs- und Reflexionsprozesse kennen und gezielt einsetzen können
- Als Teilnehmende bzw. in der Rolle als EBS: Sicherheit in ethischen Fallbesprechungen gewinnen (als Teilnehmende ohne eigenständige Moderation!) sowie Orientierung über Modelle und Methoden zur Strukturierung

Im Wirken in der Rolle als EBS zeigen diese auf den jeweiligen Stationen dann die folgenden Fähigkeiten im Sinne der Performanz:

- eine ethische Fragestellung situativ zu identifizieren und als solche zu konkretisieren
- Spannungsfelder, Asymmetrien und ethische Konfliktfelder wertfrei zu benennen
- sich in Beratungs‑, Reflexions- und Abwägungsprozesse konsensorientiert einzubringen
- Perspektiven zu wechseln und variierende Wertorientierungen zu antizipieren und zu reflektieren
- den Prozess der ethischen Entscheidungsfindung nachzuvollziehen und zu reflektieren
- den systematisierten Umgang mit ethischen Konflikten im UKT zu verorten und achtsam notwendige, entlastende und/oder unterstützende Maßnahmen anzubahnen

Der Bedarf an begleitenden und vertiefenden Weiterbildungsangeboten der Ethikbeauftragten muss sich in dem weiteren Verlauf zeigen und fordert seitens der EthiktrainerInnen in der Anfangsphase der Umsetzung ein hohes Maß an Sensibilität.

### Kollegiale Begleitung und Vernetzungstreffen der EBS

Um den EBS gerade in der Erprobungs- und Implementierungsphase des Tübinger Modells eine umfassende Unterstützung anzubieten, bieten die EthiktrainerInnen allen EBS eine dauerhafte kollegiale Begleitung über den Zeitraum eines Jahres an. Für jede Station mit EBS steht daher eine feste AnsprechpartnerIn zur Verfügung, die den Implementierungsprozess direkt begleiten kann.

Zum Erfahrungsaustausch sowie zur Vor- und Nachbereitung von Fallbesprechungen im Behandlungsteam sind monatlich stattfindende Vernetzungstreffen der EBS vorgesehen. Diese etwa 90-minütigen Zusammenkünfte werden von der Projektstelle bzw. der Gruppenleitung der Stabsstelle Qualitätsentwicklung und Pflegeberatung der Pflegedirektion organisiert und durchgeführt. Die hier gewonnenen Erkenntnisse und Erfahrungen können auf Wunsch der EBS zudem an das Klinische Ethik-Komitee weitergeleitet werden. Um eine kontinuierliche Vernetzung sicherzustellen, wird von den EBS eine monatliche Teilnahme an den Vernetzungstreffen oder eine Teilnahme am „Ethiktreff“ erwartet.

## Evaluation

Zur Evaluation des Tübinger Modells wird eine Prä-Post-Erhebung zur Wahrnehmung und Relevanz ethischer Konflikte im Stationsalltag sowie zum Erleben von moralischem Stress durchgeführt werden. Diese Erhebung ermöglicht nach der Implementierung eine Projektevaluation und soll der Qualitätssicherung des Angebots dienen. Folgende Forschungsfragen standen dabei im Fokus (vgl. Graeb 2019, S. 47–53): Wie häufig kommen ethische Dilemmata bzw. Konflikte bei Pflegekräften im Universitätsklinikum Tübingen vor und wie stark werden die Pflegekräfte dadurch belastet? Welche Faktoren wirken auf das grundsätzliche Erleben von und die erlebte Belastung durch ethische Konflikte ein? Wie wirkt sich ein fehlender Einbezug von Pflegekräften in Therapieentscheidungen auf das Konflikterleben und die Belastungen aus?

Folgende valide und reliable Instrumente wurden für die Prä-Erhebung eingesetzt: Fragebogen „Berufsethisches Verhalten und moralischer Stress“ (Kleinknecht-Dolf et al. 2015), deutsche Version adaptiert nach „Hamric’s version of Corley’s ‚Moral Distress Scale‘“ (MDS) (Kleinknecht-Dolf et al. 2017, 2015; Corley 1995; Hamric und Blackhall 2007); Fragebogen zur Wahrnehmung ethischer Konflikte im Stationsalltag sowie Belastungen und Ursachen (vgl. Sauer 2011; Graeb 2019). Dazu werden Daten zur Person und Daten zu klinischen Ethikberatungen am Klinikum erhoben (Eigenentwicklung). Somit setzt sich der Fragebogen wie folgt zusammen: i. Angaben zur Person, ii. berufsethisches Verhalten und moralischer Stress, iii. Wahrnehmung ethischer Konflikte im Klinikalltag, iv. Erfahrungen mit Ethikberatung am Klinikum.

Die Erhebung wird in allen bettenführenden Abteilungen (Stationen) im Universitätsklinikum Tübingen (Vollerhebung) bei allen Pflegekräften durchgeführt und wird zur Projektevaluation alle zwei Jahre wiederholt. Die Erhebung erfolgt anonym, auf freiwilliger Basis und ist Arbeitszeit. Vor der Datenerhebung wurde die lokale Ethik-Kommission der Fakultät informiert, ebenso die Pflegedienst- und Bereichsleitungen sowie der Personalrat. Ein Pretest wurde durchgeführt. Eine erste Erhebung auf Stationen der Ethikbeauftragten, die an der Basisqualifikation teilgenommen haben, fand von März bis Juli 2020 statt und wird derzeit ausgewertet.

## Ausblick

Im Frühjahr und Herbst 2020 wurden die ersten Basisqualifizierungen mit insgesamt 14 EBS der Intensivstationen sowie eng angrenzenden Stationen des Klinikums durchgeführt, die seither durch die EthiktrainerInnen begleitet werden. Ein intensiver Austausch und eine kontinuierliche Reflektion der laufenden Praxis zwischen EthiktrainerInnen und EBS findet in den monatlichen Vernetzungstreffen sowie in der Aufbauqualifizierung statt. Bereits im Vorfeld der ersten Schulungen im Frühjahr 2020 wurde eine Befragung der Pflegekräfte der in Phase 1 des Roll-out-Plans festgelegten Bereiche zur Projektevaluation am Klinikum durchgeführt.

Erste Erfahrungen zeigen ein überaus hohes Engagement sowie große Motivation der ausgebildeten EBS. Rückmeldungen weisen darauf hin, dass durch die Qualifizierung eine erhöhte Aufmerksamkeit und Sensibilisierung für ethische Frage- und Problemstellungen erlebt wird und dass der Austausch von Erlebtem anhand von konkreten Falldarstellungen wesentlich ist. Von besonderer Bedeutung für die Implementierung des Tübinger Modells zeigten sich zudem die eingerichtete Projektstelle, das proaktive Engagement der EthiktrainerInnen sowie die enge Zusammenarbeit mit der Stabsstelle Qualitätsentwicklung und Pflegeberatung der Pflegedirektion sowie dem Klinischen Ethik-Komitee.

Zugleich deuten erste Erfahrungen bei der Implementierung auf bestehende Limitationen hin. EBS und EthiktrainerInnen stoßen in ihren Aufgaben an ihre Grenzen, nicht nur bezüglich der erforderlichen Zeitressourcen, sondern auch bezüglich bestehender hierarchischer Strukturen. Obgleich das Ausbildungsprogramm von den TeilnehmerInnen als überaus gewinnbringend und für die eigene Ethikkompetenz als förderlich erfahren wird, können im Rahmen einer etwa viertägigen Qualifizierung der EBS nur die Grundlagen der professionellen Pflege- und Medizinethik gelegt werden. Zugleich stießen die Lehrinhalt bei den TeilnehmerInnen auf großes Interesse, welches etwa durch eine Weiterqualifizierung im Rahmen des AEM-Programms für EthikberaterInnen im Gesundheitswesen ausgebaut werden kann (AEM 2010, 2019). Um entsprechende Impulse zur Vertiefung der Ethikkompetenz der EBS zu fördern, besteht die Zusage einer Kostenübernahme des Klinikums für ein solches weiterführendes Qualifizierungsprogramm.

Wie allerorts hat die COVID-19-Pandemie das Universitätsklinikum Tübingen seit dem Frühjahr 2020 vor große Herausforderungen gestellt, sei es bei einer adäquaten Versorgung von PatientInnen unter Krisenbedingungen, oder auch durch Priorisierungsentscheidungen in Anbetracht einer drohenden Ressourcenknappheit (Marckmann et al. 2020). In dieser Krisensituation sind MitarbeiterInnen des Klinikums verstärkt mit ethischen Fragen und Problemen konfrontiert. Entscheidungen in diesen Situationen können psychisch belastend sein und werfen zugleich die Frage nach der Rolle von Ethikberatung im Rahmen der Pandemie auf (AEM 2020). Gerade auch in dieser Situation möchte das Tübinger Modell Räume schaffen, in denen die Auseinandersetzung mit ethischen Aspekten in der Klinik nicht nur in Akutsituationen erfolgt, sondern zur alltäglichen professionellen Berufspraxis wird, zur Qualität und Transparenz von Entscheidungen beiträgt und schließlich eine moralische und auch psychische Entlastung bewirkt.

## Footnotes

1 Weitere Informationen sind der Webseite des Klinischen Ethik-Komitees des Universitätsklinikums Tübingen zu entnehmen: https://www.medizin.uni-tuebingen.de/de/das-klinikum/einrichtungen/zentrale-einrichtungen/klinisches-ethikkomitee. Zugegriffen: 7. Mai 2021.

2 Zu den Mitgliedern der Unterarbeitsgruppe der AG Ethik gehörten auch Ursel Basener-Roszak und Sibylle Braun. Ihnen gebührt ein besonderer Dank für den anhaltenden produktiven Austausch.
